# Paraspinal back muscles in asymptomatic volunteers: quantitative and qualitative analysis using computed tomography (CT) and magnetic resonance imaging (MRI)

**DOI:** 10.1186/s12891-020-03432-w

**Published:** 2020-06-26

**Authors:** Eun Kyung Khil, Jung-Ah Choi, Eunjin Hwang, Sabrilhakim Sidek, Il Choi

**Affiliations:** 1 Department of Radiology, Hallym University College of Medicine, Hallym University Dongtan Sacred Heart Hospital, 7, Keunjaebong-gil, Hwaseong-si, Gyeonggi-do 18450 South Korea; 2 Medical Imaging Unit, Faculty of Medicine, Universiti Teknologi MARA (UiTM) Sg Buloh Campus, 47000 Sg, Buloh, Selangor Malaysia; 3 Department of Neurosurgery, Hallym University Dongtan Sacred Heart Hospital, 7, Keunjaebong-gil, Hwaseong-si, Gyeonggi-do 18450 South Korea

**Keywords:** Sarcopenia, MRI, Cross-sectional area, Fatty infiltration, Muscle atrophy, Two point Dixon, Fat fraction, Goutallier score, Paraspinal muscle

## Abstract

**Background:**

To evaluate paraspinal back muscles of asymptomatic subjects using qualitative and quantitative analysis on CT and MRI and correlate the results with demographic data.

**Methods:**

Twenty-nine asymptomatic subjects were enrolled prospectively (age: mean 34.31, range 23–50; 14 men, 15 women) from August 2016 to April 2017. Qualitative analysis of muscles was done using Goutallier’s system on CT and MRI. Quantitative analysis entailed cross sectional area (CSA) on CT and MRI, Hounsfield unit (HU) on CT, fat fraction using two-point Dixon technique on MRI. Three readers independently analyzed the images; intra- and inter-observer agreements were measured. Linear regression and Spearman’s analyses were used for correlation with demographic data.

**Results:**

CSA values were significantly higher in men (*p* < 0.001). Fat fraction was higher (22.53% vs. 14.35%) and HU lower (36.00 vs. 47.43) in women (*p* < 0.001). Intra- and inter-observer reliabilities of the two methods were greater than 0.8, except for CSA of L5/S1 on MRI; however, regarding quantitative analysis, decreasing HU and increasing fat fraction were correlated with increasing age, female gender and lower lumbar segment (*p* < 0.001).

**Conclusion:**

MRI and CT can be reliably used for qualitative and quantitative analysis of paraspinal back muscles, regarding fat content. Fat fraction and HU showed highest reliabilities.

## Background

Atrophy and degenerative changes in the back muscles are known to be associated with chronic lower back pain [1, 2]. Studies have been conducted to investigate the relationship between low back pain, sarcopenia, and related pathologies [1, 3–5]. However, there have been few studies on analysis of paraspinal back muscles in asymptomatic persons [6–10] and fewer studies in young adults [1, 6, 10]. Studies of lumbar paraspinal muscles are divided into two categories: qualitative and quantitative analysis by cross-sectional area (CSA). For qualitative analysis, Goutallier grade (GG) system has been used extensively in CT and MRI studies of rotator cuff muscles [10–12]. However, there has been no standardized grading system to evaluate degenerative changes of paraspinal muscles in patients; several studies have applied the GG system to lumbar paraspinal muscles [2, 6, 10, 11, 13]. Lumbar paraspinal muscles are composed of various muscles and degree of fatty change varies according to lumbar level ^18^; therefore, GG system has to be further validated in application to paraspinal muscles.

Some quantitative studies of paraspinal muscles have used the ratio of CSAs on CT or MRI [3, 5, 14–17]. However, it may be difficult to generalize CSAs to represent degenerative changes in lumbar paraspinal muscles because of differences in people’s body composition. There have been studies evaluating functional CSA (fCSA), measuring areas without fatty changes, or total CSAs of the paraspinal muscles [3, 7, 16–18]. Other studies have used various fat quantification techniques to overcome shortcomings of quantitative techniques, including MR spectroscopy and chemical shift [1, 2, 4, 6, 10, 19, 20]. We used the two-point dixon technique because fat fraction (FF) can readily be obtained on a clinical scanner within reasonable time [21].

Although there have been several studies evaluating quality of lumbar paraspinal muscles, few studies have used CT and MRI comparing qualitative and quantitative methods [18]; fewer studies evaluated all lumbar segments. Therefore, in this study, we analyzed the lumbar paraspinal muscles both quantitatively and qualitatively on CT and MRI at all lumbar segments in young asymptomatic adults, regarding reliability of these methods and analyzed the correlation with demographic variables, especially regarding fat content.

## Methods

### Study population

This was a prospective study of asymptomatic healthy volunteers (age: mean 34.31, range 23–50; 14 men, 15 women) from August 2016 to April 2017; they were recruited from a health screening program, which usually gives the choice of having CT or MRI performed upon the subjects’ choice; Institutional Review Board approval and written informed consent were obtained. Exclusion criteria were previous procedure and/or surgery of spine, hip, or knee, poliomyelitis or congenital anomalies in spine or lower extremity, and contraindications for MRI. Twenty-nine subjects were enrolled, clinically examined, and measured for height and weight with body mass index (BMI) as weight/height^2^ (kg/m^2^); all subjects underwent lumbar CT and MRI according to standardized protocols.

### CT and MR imaging

Axial and sagittal reformatted images were acquired on a multidetector CT scanner (Somatom Definition AS or Somatom Definition Flash, Siemens, Erlangen, Germany) from T12 upper endplate margin to S2 lower endplate margin. CT scanning parameters were as follows: 100 to 120 kV, 250–750 mAs, 0.6 mm collimation, and 2 mm slice thickness. MRI was performed using a 3.0 T scanner (Skyra; Siemens, Erlangen, Germany). T2-weighted FSE (fast spin echo) axial and sagittal, T1-weighted axial sequences were acquired from L1–2 to L5-S1 centered at each intervertebral disc. Additionally, axial two-point Dixon sequence was obtained parallel to each vertebra (Table 1).

**Table 1:** Parameter of L-spine MRI protocol

Parameters	T2 weighted Sagittal	T1 weighted Sagittal	T2 weighted Axial	T1 weighted Axial	Two point Dixon
Repetition time (ms)	3040	504	2830	425	5.36
Echo time (ms)	91	9.8	100	15	2.46–3.69
Matrix number	410*512	269*448	230*384	230*384	126*224
Field of view (mm)	320	320	160	160	196
Number of acquisition	2	1	2	2	1
Echo train length	19	5	16	3	X
Section thickness (mm)	3	3	4	4	3
Intersection gap (mm)	0.6	0.6	0.4	0.4	0.6
Bandwidth (kHz/pixel)	257	248	246	241	890
Acquisition time (min: sec)	1:54	1:30	1:48	2:01	0:15
Flip Angle	160	130	130	125	9

### Image analysis

**Fig. 1:**
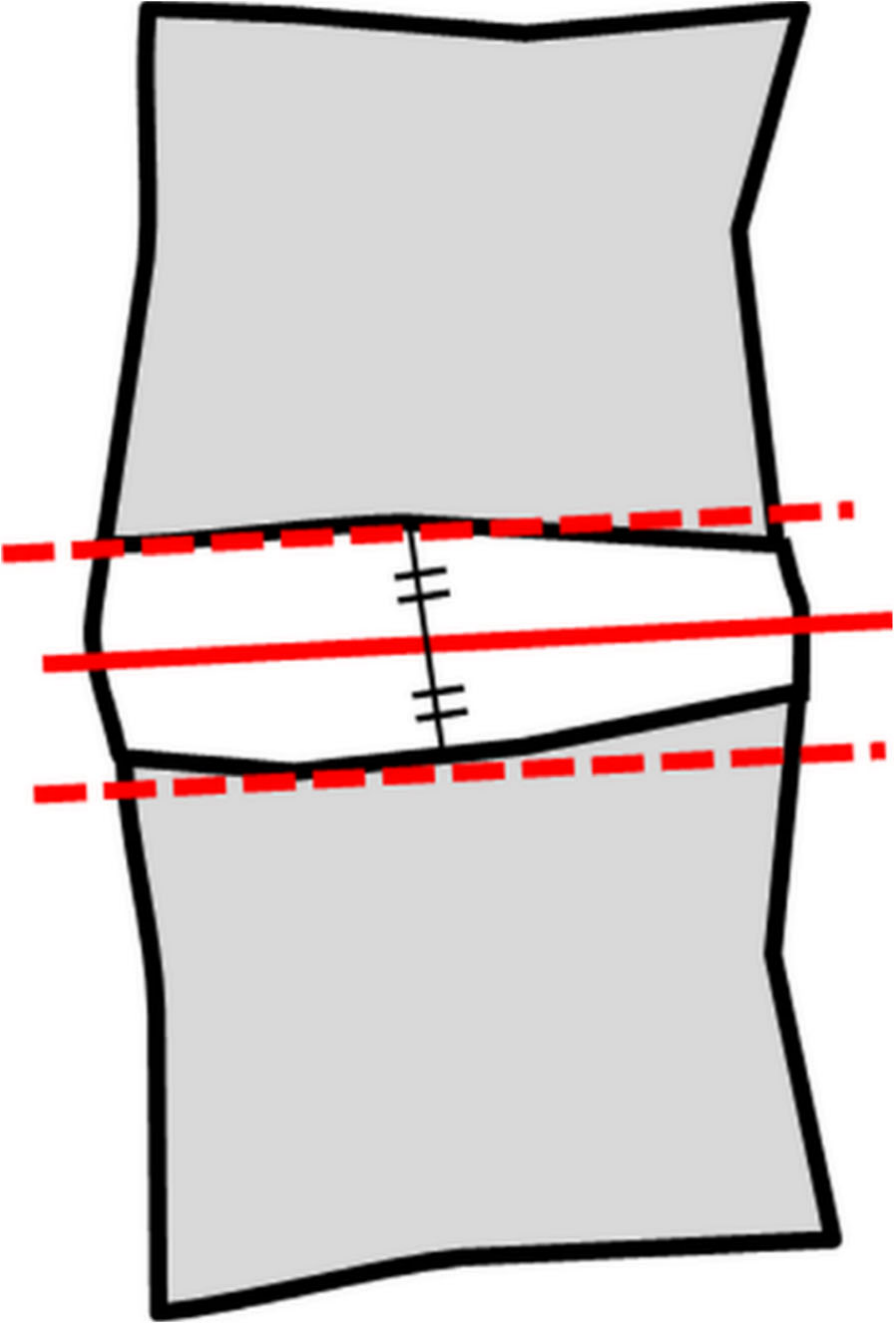
A schematic diagram defining an axial plane in sagittal plane of CT and MRI. The axial plane was obtained by looking at the point parallel to the disc passing through the center of the disc height. And consecutive images were obtained according to CT and MR parallel to the central line

**Fig. 2:**
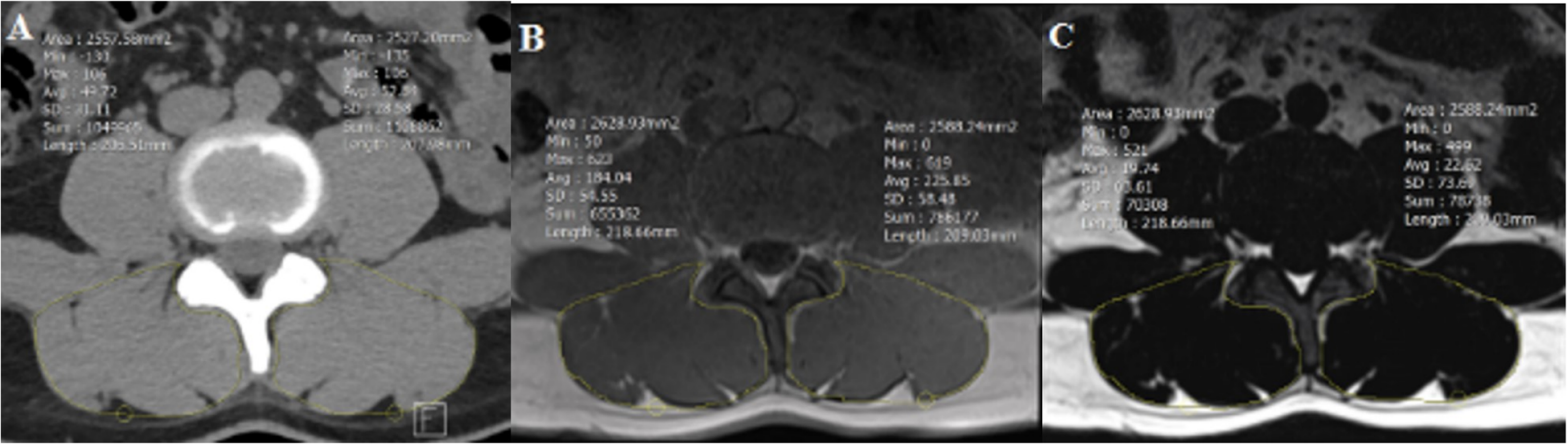
Image shows an example of the region of interest (ROI) of the paraspinal back muscle on axial plane and acquisition of the quantitative measurement values on PACS workstation. On CT (**a**), when the ROI is drawn, mean CSA (cross sectional area) and mean HU (Hounsfield unit) are displayed on screen. On MRI (**b**), when the ROI is drawn on in-phase image of two-point Dixon technique, the CSA and signal intensity values are displayed as on CT, and the ROI is copied and pasted onto the fat only image (**c**), then CSA and signal intensity measured

Two musculoskeletal radiologists (reader 1 with six-year experience, reader 3 with four-year experience) and a trainee (reader 2) independently analyzed images on Picture archiving and communicating system (PACS; Infinitt Co., Ltd., Seoul, Korea). We obtained axial images parallel to intervertebral discs on CT and MRI and selected an axial plane passing through the disc center (Fig. 1). On axial CT and MRI, regions of interest (ROIs) were manually drawn along the thoracolumbar fascia (Fig. 2); this corresponded to the “total CSA” referred to in previous studies, not “fCSA” [9, 16]. We assessed inter- and intraobserver reliabilities of qualitative and quantitative measurements on CT and MRI. First, the three readers were trained by one experienced observer how to draw ROIs and measure GGs under consensus and analyzed images blinded to each other’s results. Measurements were repeated after 2 weeks for intraobserver agreement. We noted the presence of disc pathologies if there were any.

### Qualitative analysis

**Fig. 3:**
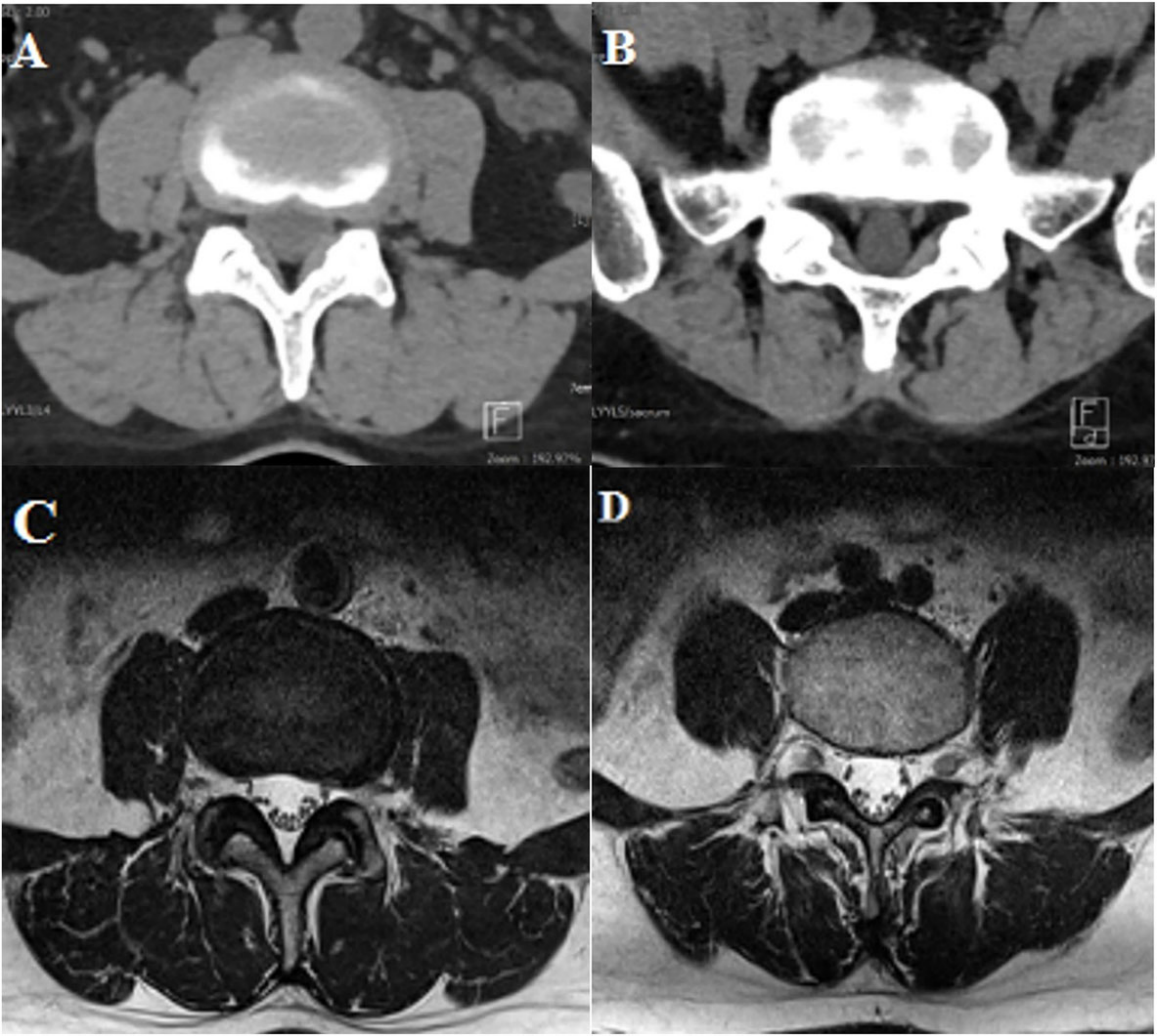
An example of Goutallier grading system on CT (**a**, **b**) and MRI (**c**, **d**) in a 49-year old woman. **a** grade I: low density fat is observed around the vertebral lamina. **b** grade II: The fat surrounding three borders of the multifidus muscle is observed, and there was atrophy change. **c** grade I: In the axial T2-weighted image, there is fatty streak in the paraspinal muscles and fat surrounding the lamina. **d** grade II: on axial T2-weighted images, the amount of fat in the muscle is increased and accompanied by atrophic change

Paraspinal muscles within the thoracolumbar fascia were evaluated using GGs on CT and MRI at L1–2 to L5-S1 segments as follows: 0 - all muscle, no fat, 1 – fatty streaks within muscle or fat stripe around lamina and facet joint, 2 - more muscle than fat, 3 - muscle equal to fat, 4 - more fat than muscle (Fig. 3).

### Quantitative analysis

On CT, three radiologists drew ROIs on axial images to measure Hounsfield units (HU) and CSAs (Fig. 2a). On MRI, in-phase and fat only image were obtained using two-point Dixon method with ROIs drawn on in-phase image (Fig. 2b), which were copied and pasted onto fat only image (Fig. 2c); CSA and mean signal intensity (SI) were measured. FFs were calculated by dividing SI of the fat image ROI by SI of the in-phase image ROI. Additionally, on both CT and MRI, the values ​​were corrected using the ROI of the vertebral body (VB) to reduce bias such as body size and gender that could affect the CSA. The ROI was drawn along the margin of the VB at the inferior endplate of each lumbar level and then divides the measured CSA by this value [19, 22].

### Statistical analysis

Demographic data were analyzed using T-test for parametric variables and Mann-Whitney U-test for non-parametric variables. For qualitative analysis, Kappa statistic was used for intra-observer agreement, Kendall’s coefficient of concordance for inter-observer agreement. For quantitative analysis, intra-class correlation coefficient (ICC) was used for intra- and inter-observer agreements. Since GGs of paraspinal muscles were analyzed at 5 lumbar segments, it was impossible to obtain one representative value. We evaluated the relationship between demographic variables, including age, sex, BMI, and quantitative values. Also, we analyzed associations between quantitative values and each lumbar segment, i.e. whether there was an increase according to lumbar segment. Associations were analyzed using simple linear regression analysis; additional multiple linear regression analysis was performed for age, sex, and BMI, regarding their influence. Relationship between GG and quantitative values were analyzed using simple linear regression. Spearman’s correlation analysis was used to evaluation of correlation between mean FF/HU and each lumbar segment, and then determine which lumbar segment best reflected the mean FF and HU. Kappa values can be interpreted as follows: under 0.20 slight agreement, 0.21–0.40 fair agreement, 0.41–0.60 moderate; correlation coefficient (r) indicated the degree of relevance as follows: 0.2–0.4 week, 0.4–0.7 moderate, 0.7–0.9 strong, over 0.9 very strong. Statistical analyses were performed using SPSS 20.0 software (SPSS Inc., Chicago, IL, USA) with significance for *P* < .05.

## Results

Regarding demographic data, height, weight, and BMI were significantly higher in males as expected (Table 2). Upon analysis of incidental disc pathologies, the results were as follows: there 5 disc protusions in L3/4, L4/5, L5/S1 and no disc extrusion. Qualitative and quantitative values of paraspinal muscles of each lumbar segment are summarized (Table 3). GGs scored between 0 and 2 on CT and MRI. Mean GG increased down the lumbar segments and was higher in women; GGs on MRI were higher than CT in both genders.

**Table 2:** Descriptive statics of demographics data for 29 healthy volunteers

	Mininum	Maximum	Mean	Standard deviation	Men (*n* = 14)	Women (*n* = 15)	*p*-value
Age (year)	23.00	50.00	34.31	8.59	33.14 ± 6.83	35.40 ± 10.09	0.81*
Height (cm)	155.00	186.00	169.91	10.18	178.58 ± 6.03	161.81 ± 5.31	< 0.001
Weight (kg)	49.00	94.00	66.97	13.52	77.41 ± 9.41	57.22 ± 8.51	< 0.001
BMI (kg/m^2^)	19.30	29.40	22.98	2.64	24.27 ± 2.66	21.77 ± 2.01	0.008
Fat fraction (%)	9.69	29.25	18.44	6.39	14.35 ± 3.68	22.53 ± 5.93	< 0.001
HU	25.93	53.45	41.52	7.92	47.43 ± 4.30	36.00 ± 6.38	< 0.001
MR CSA (mm^2^)	1440.38	2921.77	2013.29	403.08	2297.59 ± 282.26	1729.00 ± 289.29	< 0.001
CT CSA (mm^2^)	1529.25	3436.73	2172.38	479.15	2519.67 ± 404.06	1848.24 ± 272.74	< 0.001

Using t-test, *using Mann-Whitney U test; *BMI *Body mass index, *HU* Hounsfiled unit, *CSA* Cross sectional area

**Table 3:** The qualitative and quantitative values of the paraspinal muscles in each lumbar segment

L-spine level	Men								Women							
	CT				MRI				CT				MRI			
	GG	CSA of PM (mm^2^)	CSA ratio	HU	GG	CSA of PM (mm^2^)	CSA ratio	Fat fraction (%)	GG	CSA of PM (mm^2^)	CSA ratio	HU	GG	CSA of PM (mm^2^)	CSA ratio	Fat fraction(%)
L1/2	0.00*	2475.52	171.64*	52.55	0.30*	2464.85	197.43*	8.15	0.07*	1608.45	139.07*	45.91	0.74*	1634.75	161.21*	13.33
L2/3	0.20	2587.25	168.86*	51.46	0.53	2635.92	191.29*	10.17	0.29*	1827.64	144.6*	42.24	0.93*	1851.84	165.07*	15.77
L3/4	0.23*	2687.26	165.36*	47.96	0.83*	2624.13	175.57	13.52	0.75	1953.68	147.73*	38.32	1.11	1968.90	161.93	21.07
L4/5	1.00	2651.20	159.71	44.66	1.37	2419.63	164.36	16.88	1.43	2090.03	160.47	30.65	1.17	1971.08	171.64	27.63
L5/S1	1.40*	2197.15*	143.51	40.53	1.87*	1343.42*	101.86*	23.01	1.82	1764.43*	141.25	22.89	1.89	1218.41*	121.21*	34.84
Total	0.57	2519.67*	387.36*	47.43	0.98	2297.59*	166.14	14.35	0.87	1848.24*	299.71*	36.00	1.27	1728.99*	156.22	22.53

*GG* Mean Goutallier grade, *CSA* Cross sectional area, *PM* Paraspinal muscle, *CSA ratio* Paraspinal muscle CSA/vertebral body CSA*100, *HU* Hounsfield unit

In GG (between both men and women), ‘*’ means *p* < .05 using Mann-Whitney U test

In CSA, CSA ratio (between both men and women), ‘*'means *p* < .05 using T-test

Mean CSA of men and women were 2519.67 mm^2^/2297.59mm^2^ and 1848.24 mm^2^/1729.00mm^2^ on CT/MRI, respectively: mean CSA and the CSA ratio of men were mostly significantly higher than women on CT/MRI. CSAs decreased at lower segments. CSAs on CT and MRI were comparable, except for L5-S1 level. Mean HU values were 47.43(±4.30) in men and 36.00(±6.38) in women; HU values decreased at lower levels. Mean FF was 14.35%(±3.68) in men and 22.53%(±5.93) in women; FFs increased at lower segments. On analysis of incidental disc pathologies and degenerative changes of 145 lumbar segments, 6 disc protrusions and 11 bulging discs were found, however, all asymptomatic.

### Intra- and inter-observer reliability

For intraobserver reliability, regarding qualitative analysis on CT, in reader 1, there was substantial to almost perfect agreement (Kappa value 0.78–0.84) in all levels except for L1–2 level. In readers 2 and 3, Kapppa values were 0.74–1.0 and 0.76–1.0, respectively. On MRI, values of reader 1, 2, and 3 ranged between 0.78–1.0, 0.73–0.93, and 0.81–1.0, respectively. Regarding quantitative analysis, Kappa values were higher than 0.9 for FF and HU in all readers. For CSAs, values were 0.9 or higher at all levels, except for L5/S1 level on MRI in all readers.

For inter observer reliability, ICCs for GGs were 0.83–0.92 and 0.87–0.93 for CT and MRI, respectively. ICCs for CSA on CT were 0.95–0.97, on MRI were 0.94–0.98 at levels L1–2 to L4–5; the lowest ICC was 0.61 at level L5-S1. For HU and FFs on CT and MRI, ICCs were 0.9 or higher at all lumbar segments.

### Association analysis

Mean quantitative values were analyzed regarding the relationship between age, sex, and BMI; the influence of each variable was measured by simple regression analysis (Table 4). CSAs showed no significant difference according to lumbar segment on CT but significantly decreased down lower segments on MRI (*p* < 0.001). HU decreased and FF significantly increased down lumbar segments (*p* < 0.001). Additionally, we analyzed correlation between qualitative and quantitative values; right and left side values were comparable. As GGs increased, CSA decreased on CT and MRI, respectively, which was significant on CT (*p* = 0.002) but not on MRI. However, as GGs increased, HU decreased and FF increased (*p* < 0.001).

**Table 4:** The relationship between quantitative analysis methods and age, sex, MRI, L-spine level, and the relationship between quantitative and qualitative methods by linear regression analysis

Variable	CSA (mm^2^)						HU			Fat fraction (%)		
	CT			MRI			CT			MRI		
	B	SE	*p-value*	B	SE	*p-value*	B	SE	*p-value*	B	SE	*p-value*
Age	−6.00	10.67	0.578	−2.72	9.08	0.767	−0.51	0.15	0.002	0.45	0.11	0.001
Sex												
male	1			1			1			1		
female	− 671.43	127.21	< 0.001	− 568.59	108.02	< 0.001	−11.43	2.04	< 0.001	8.18	1.86	< 0.001
BMI	147.77	20.38	< 0.001	120.46	19.41	< 0.001	1.10	0.54	0.050	−0.87	0.45	0.065
L-spine level	5.61	31.28	0.858	− 158.63	32.58	< 0.001	−4.47	0.52	< 0.001	4.57	0.42	< 0.001
Goutallier grade												
Right	− 164.72	51.28	0.002	− 334.02	56.45	0.229	−11.21	0.67	< 0.001	6.13	0.87	< 0.001
Left	− 178.81	56.85	0.002	−345.25	60.74	0.143	−10.98	0.65	< 0.001	6.13	0.87	< 0.001

*B* Linear regression coefficient, *SE* Standard error, *CSA* Cross sectional area, *HU* Hounsfield unit

Regarding CSAs on CT and MRI, there were significant correlations with sex and BMI (Table 5). CSA was smaller in women and as BMI increased, CSA significantly increased on CT and MRI (*p* < 0.001). HU and FF were statistically correlated with age and sex but not with BMI. Lower HU and higher FF were observed in women (*p* < 0.001). Gender was the most influential variable for HU and FF; BMI was the most influential variable for CSA.

**Table 5:** The association analysis between the quantitative analysis methods and three variables by multiple linear regression

Variable	CSA (mm^2^)						HU			Fat fraction (%)		
	CT, Adj R^2^ = 0.779			MRI, Adj R^2^ = 0.765			CT, Adj R^2^ = 0.730			MRI, Adj R^2^ = 0.663		
	B(β)	SE	*p-value*	B(β)	SE	*p-value*	B(β)	SE	*p-value*	B(β)	SE	*p-value*
Age							−0.43 (−0.46)	−0.09	< 0.001	0.38 (0.519)	0.08	< 0.001
Sex	− 393.79 (− 0.42)	95.56	< 0.001	− 365.32 (− 0.46)	86.23	< 0.001	−10.47 (− 0.67)	1.54	< 0.001	7.14 (0.569)	1.42	
BMI	111.14 (0.61)	18.44	< 0.001	87.56 (0.56)	16.98	< 0.001						

*B* Linear regression coefficient, *β* Standardized linear regression coefficient, *SE* Standard error, *Adj R*^*2*^ Adjusted R square, *CSA* Cross sectional area,* HU* Hounsfield unit, *BMI *Body mass index

When each lumbar level was analyzed for the most representative FF and HU, FF at L3–4 was the most representative in all readers; FF showed a strong correlation with r value of 0.9 or more, except at L5-S1 level. HU on CT showed the highest values at levels L2–3 and L4–5, with r values of 0.9 or higher.

## Discussion

Muscle degeneration with fatty change is associated with lumbar spine pathology affecting disease progression and life quality [5, 15, 20, 23]. Although CT and MRI are known to be accurate and sensitive in muscle evaluation [24, 25], there has been a few studies on paraspinal muscles in asymptomatic young adults using both modalities [6–9, 19]. As MRI techniques have evolved recently, validation and comparison in healthy adults are needed. In our study, subjects were relatively young adults, matched by gender with a mean BMI of 23.0 kg/m^2^.

GGs ranged between 0 and 2 in our study, consistent with previous studies [2, 10], increasing down lower lumbar segments, showing highest degree of fatty degeneration in L5-S1 segment, consistent with previous studies [26]. Although GGs correlated with quantitative analysis, we found a limitation in applying GGs to evaluate back muscles of young adults since higher degree of muscle degeneration (grades 3, 4) were not seen and fatty changes were seen only in parts of paraspinal muscles close to spine; in spite of this, interobserver agreement of GGs was high.

GGs showed statistically significant differences between genders on CT and MRI; scores on MRI tended to be higher because MRI is more sensitive in detecting fat [19, 20, 27]. However, regarding CSA values, there was no statistically significant difference between CT and MRI, except for L5-S1 segment. Axial images using two-point Dixon technique were acquired parallel to each vertebra unlike conventional axial images. The angle between L5 and S1 vertebral bodies was large, and so CSA on two-point Dixon sequence and axial plane of CT were measured differently, resulting in lower interobserver agreement at L5-S1 segment on MRI.

CSAs were significantly higher in men on CT and MRI but did not show a significant difference according to age, consistent with previous studies [6, 14] and contradictory to other studies hypothesizing increased degenerative muscle changes with aging [15, 23]. This may be due to our study population, consisting of relatively young asymptomatic volunteers. Increased CSA was strongly associated with increasing BMI. As CSA does not accurately reflect muscle quality in young adults, BMI has limitations in assessing muscle quality. In our study, association of BMI with HU or FF was not statistically significant. Only quantifying fat could accurately evaluate the quality of paraspinal muscles.

HU and FF data were consistent with the hypothesis that fatty degeneration of paraspinal muscles increases with age [6, 15]; fatty change tended to increase down lower segments, more pronounced in women, consistent with previous studies [20, 28]. Fortin et al. [16] proposed the most abundant fatty change to occur at L5-S1 segment because the gravitational centerline passes through this segment rendering it to be the most weight-bearing; also, a larger lordotic angle and greater motion at this level may contribute to this finding [16].

Previous studies have attempted to find the lumbar level, most representative of the mean [6]; Crawford et al. [6] noted L4–5 segment to be the most representative FF on MRI. We expected that the mid-lumbar level would best reflect fatty changes, because fatty change increased down lumbar levels, reflected in decreasing HU and increasing FF. From our results, all lumbar segments seemed to reflect the mean value well on CT and MRI. This may be because the study included only asymptomatic adults and difference in the correlation coefficient among the levels was not significant. The level that best reflected the mean was L2–3 segment for HU and L3–4 segment for FF; correlation coefficient was lower at L5-S1 segment than other levels. We hypothesized a fatty change with muscle atrophy in the lower lumbar segments, most prominent in L5/S1. Similar to our study, a 15-year prospective study of quantitative analysis of paraspinal muscles also showed greater atrophy and fatty changes in muscle at L5/S1 [16].

Regarding qualitative analysis, intra- and interobserver agreements for CSA were high, consistent with previous studies [9, 18], confirming CSA reliable for muscle evaluation. Also using FF and HU, more reliable values were obtained, comparable to CSA. Recently, two-point Dixon technique has been used to quantify fat in paraspinal muscles [6]. Since two-point Dixon technique acquires four phases with one acquisition, images are obtained in a reasonable scan time. In addition, on CT, it is easy to measure the degree of fatty change using only HU. Therefore, FF and HU are feasible and reliable tools for evaluation of muscle quality, especially regarding fatty degeneration.

This study has several limitations. First, sample size was small and did not include a wide age range. However, we obtained data of lumbar paraspinal muscles in asymptomatic relatively young adults, which have not been studied much. Secondly, this study reflects the characteristics of a certain ethnicity and may not reflect a diverse population. Thirdly, although asymptomatic healthy adults were recruited, there were some disc pathologies; however, they were all asymptomatic and probably did not have a significant effect on our results. Fourthly, there was no gold standard for muscle degeneration; however, it is neither feasible nor ethical to obtain muscle tissue specimens in a study like this and lack of gold standard is exactly the reason for such studies. Finally, we did not subdivide muscle groups; we analyzed all muscles in the thoracolumbar fascia as a whole as this seemed to be a simple and easy method.

## Conclusion

Female, older age, and lower lumbar segment were associated with higher fat content of paraspinal muscles. MRI and CT can be reliably used for qualitative and quantitative analyses of paraspinal back muscles in young healthy adults, especially regarding fat content, with good correlation between the two methods. FF and HU could be useful tools for evaluating muscle degeneration with fatty change in paraspinal muscles. The level that best reflected the mean was L2–3 segment for HU and L3–4 segment for FF. This study could serve as a baseline study for future studies regarding muscles.

## Data Availability

The dataset analyzed are not publicly available but are available from the corresponding author on reasonable request.

## Abbreviations

CT: Computed tomography
MRI: Magnetic resonance imaging
CSA: Cross sectional area
HU: Hounsfiled unit
GG: Goutallier grade
fCSA: Functional cross sectional area
FF: Fat fraction
BMI: Body mass index
FSE: Fast spin echo
ROIs: Regions of interests
SI: Signal intensity
ICC: Intra-class correlation coefficient
